# Comparison of serum vitamin D levels between healthy and ADHD children

**DOI:** 10.22088/cjim.14.4.681

**Published:** 2023

**Authors:** Armon Massoodi, Sakineh Javadian Koutanaei, Zahra faraz, Zahra Geraili, Seyedeh Maryam Zavarmousavi

**Affiliations:** 1 Social Determinants of Health Research Center, Health Research Institute, Babol Univrsity of Medical Sciences, babol, Iran; 2 Department of Psychiatry, School of Medicine, Social Determinants of Health Research Center, Research Institute for Health, Shahid Yahyanejiad Hospital, Babol University of Medical Sciences, Babol, Iran; 3 Student Research Committee, Babol University of Medical Sciences, Babol, Iran; 4 Department of Psychiatry, Shafa Hospital, Gilan University of Medical Sciences, Rasht, Iran

**Keywords:** Attention-deficit/hyperactivity disorder, Hyperactivity disorder, Vitamin D, Psychiatric disorders.

## Abstract

**Background:**

The most common psychiatric disorder in childhood is Attention-deficit/hyperactivity disorder (ADHD). Researchers have studied the effects of micronutrients on ADHD in recent years, but vitamin D (vit D) deficiency has received less attention. In this study, serum vit D levels were compared between healthy and ADHD children.

**Methods:**

This case-control study was carried out, in 2020, on 6-to-12-years-old children. There were 45 children with ADHD in the case group and 45 healthy children in the control group. Intravenous blood samples were taken from each child to measure serum vitamin D levels. A p-value < 0.05 was considered as significant.

**Results:**

Mean serum vit D levels in children with ADHD (17.34±8.37 ng / ml) were significantly lower than those in the control group (23.02±10.97 ng / ml) (P= 0.007). There were no significant differences in mean serum levels of vit D due to ADHD subtypes. Mean serum vit D levels were not significantly associated with the gender of children with ADHD. There was an inverse correlation between vit D levels and the severity of ADHD, but it was not statistically significant.

**Conclusion:**

The present study showed that children with ADHD had significantly lower serum vit D levels than healthy controls.

Attention-deficit/hyperactivity disorder (ADHD) is an early-onset chronic nervous system-related disorder characterized by attention deficit, hyperactivity, and impulsivity (1). It affects 2-9% of children and adults (2), and is one of the most common psychiatric disorders in childhood (3). ADHD usually begins in childhood and often lasts for life (4). The disease can affect the performance of affected children, other family members, peers, and teachers (5). Childhood learning can be influenced by ADHD and can cause various psychosocial problems in children and adults (6). These people are generally less educated than healthy people, have fewer professional and social skills to acquire in adulthood, and can put significant health and financial burdens on society (7, 8). In addition to the three main symptoms, other concomitant symptoms such as violence, social skills deficiencies, conflict with peers, and behavioral problems are also important symptoms (9, 10). Although the etiology of ADHD is not fully understood, different environmental elements (exposure to specific foods or inhalants) and genetical factors have been suggested as the etiology of the disease (11). Vitamin D (Vit D) is indeed effective in brain development, especially in the early stages of fetal and infancy. Vit D is required for brain development through various mechanisms, and fetal and childhood vit D deficiencies affect brain structure and function (12). 

Vit D deficiency has been linked to anxiety, mood, and developmental disorders in some studies (13–15). Vit D has also been reported to affect brain growth and act as a neuro-immuno-modulator for behavioral and psychiatric disorders (13) and (14). The lack of vit D reduces tissue differentiation and decreases the expression of neurotrophic factors in animal studies (15). In addition, clinical trials have reported that such changes in the brain manifest themselves as abnormal behavioral changes and excessive movement in these animals (16). It has been founded that the enzymes 1α-hydroxylase (vit D activating enzyme) and vitamin D receptors are widely distributed in the substantia nigra, hippocampus, hypothalamus, and prefrontal cortex which are parts of the brain. Many of these areas are associated with the pathogenicity of ADHD in the brain (5, 17). 

There are several mechanisms by which vit D supplements reduce the symptoms of ADHD. Vit D is essential for the production of the neurotransmitters dopamine and norepinephrine, which alleviate the symptoms of ADHD (5). Vit D has also been described as a hormone and an important regulator of serotonin production, which plays an important role in brain function. Adults with ADHD have been shown to display more aggressive behavior by decreasing serotonin levels in their brains. Serotonin-producing genes such as tryptophan hydroxylase-2 and others may be associated with higher sensitivity to ADHD at the molecular level. In adult mice with enzyme gene polymorphisms, vit D deficiency may be associated with vit D status, as the Tph2 gene is activated by vit D. Restricting vitamin D intake led to serious cognitive-behavioral problems in these mice (11).

There are many problems and costs associated with ADHD for children, families, and communities, which is why it is important to examine the underlying factors that contribute to its occurrence and development. As vit D is one of the most important nutrients for brain growth and development, its deficiency may contribute to ADHD pathogenesis, but in recent years, micronutrients have been studied for their effects on ADHD, but vit D has received less attention, so this study aimed to compare vitamin D levels between ADHD and healthy children.

## Methods

**Study Population: **In this cross-sectional analytical study, 90 children aged 6-12 years were selected as study samples at the Pediatric and Adolescent Psychiatric Clinic at Yahyanejad Hospital in Babol. Inclusion criteria included consent to participate in the study and age 6-12, and exclusion criteria were any malabsorption syndrome, neurological disorders such as seizures and metabolic disorders, serious psychiatric disorders such as mood and psychotic disorders, kidney disease, liver disease and a recent history of vit D supplementation. Children with ADHD medical records who attended a pediatric psychiatric clinic at Shahid Yahyanejad hospital were included in the case group (n=45) and non-ADHD children referred to the Babol Social Security clinic for weight and height checks were included in the control group (n=45).

**Data Collection: **Connors Parent Questionnaire and clinical interview with an adolescent and pediatric psychiatrist were used in this study to diagnose ADHD (18, 19). Pediatric and adolescent psychiatrists conducted clinical interviews with children and their parents to diagnose and assess the severity of disease symptoms in the children participating in the study (6, 20, 21). 

This study was approved by the Ethics Committee of Babol University of Medical Sciences (IR.MUBABOL.REC.1399.015). Vit D serum level of all participants was measured in the summer season. Informed consent was obtained from each participant’s parent, and 5 milliliters (ml) of venous blood were drawn from each child in the control group and the cases group, and 25-hydroxyvitamin D levels were determined in a laboratory at Shafa Babol using the Euroimmun kit with specifications (EXP: 2020/11/14 and lot number: E 191202 BO). A pathologist interpreted the results. Serum vit D levels are classified into four categories: normal (30–100 ng/ml), deficiency (10–29 ng/ml), severe deficiency (10 ng/ml), and toxic (> 100 ng/ml) (22, 23).

**Data Analysis**: The data were analyzed using SPSS V.22. Chi-square, t-tests, and Pearson correlation coefficients were used. A p-value of less than 0.05 was considered significant.

## Results

In the present study, 90 children with inclusion criteria (45 in the control group and 45 in the case group) participated. In this study, 59 (65.6%) of the participants were males, and 31 (34.4%) were females. The mean age of the children surveyed was 8.63 ± 1.83 years (range 6-12 years).

There was no significant difference between the two groups in terms of age (P=0.121) and gender (P=0.267). The mean serum vit D levels of the children who participated in the study were 20.18 ± 10.11 (1.30-57.5) ng/ml. Table 1 shows the serum levels of vit D in both groups. Further analysis of the data showed that the mean serum vit D levels of the ADHD group were significantly lower than those of the control group (P = 0.007). Table 2 shows the serum vit D levels for the two groups according to the degree of deficiency. As mentioned, 33.33% of the control group and 6.66% of the case group had normal vit D levels. 

As shown in table 3, in this study, the combined subtype had the highest frequency, and the subtype with predominant symptoms of attention deficit and hyperactivity was found to be second and third in frequency, respectively data analysis revealed no significant association between serum vit D levels and the ADHD subtype. Based on data analysis, gender did not significantly affect serum vit D levels in either group. There was a significant and negative correlation between vit D levels and ADHD symptoms (P=0.169 and r=-0.146) based on Pearson's correlation test. 

**Table 1:** The comparison of participants' serum vitamin D levels in two groups

**Serum Vitamin D (ng / ml)**				**group**
**The lowest level of vitamin D**	**The highest level of vitamin D**	**Standard deviation**	**mean**	
1/3	37	8/37	17/34	**case**
6	57/5	10/97	23/02	**control**
0.007				**p-value**

*ADHD: Attention-deficit/hyperactivity disorder

**Table 2:** Distribution of serum vitamin D levels by ADHD*

**group**	**vitamin D**			
	**Severe deficiency ** **(ng/ml 10>)**	**Mild deficiency ** **(10-29 ng/ml)**	**Normal** **(30-100 ng/ml)**	**Toxic** **(>100 ng/ml)**
**case**	(22/23%) 10	(71/11%) 32	(6/66%) 3	0
**control**	(8/88%) 4	(57/77%) 26	(33/33%) 15	0

*ADHD: Attention-deficit/hyperactivity disorder.

**Table 3:** The comparison of serum vitamin D levels, age, and gender by ADHD subtype in 6-12-year-old children in Babol

**Variable**		**subtype with predominant symptoms of hyperactivity**	**Subtype with predominant symptoms of attention deficit**	**Combined subtype**	**P-value**
**Gender**	**Male**	(80%) 4	(51.7%) 4	(72.7%) 24	0.651
	**Female**	(20%) 1	(42.9%) 3	(27.3%) 9	
	**Total**	(11.11%) 5	(15.5%) 7	(73.3%) 33	
**Age** **(Year)**	**Mean**	8	7.57	8.55	0.079
	**Standard deviation**	1.58	0.787	1.66	
**Vitamin D** **)** **ng/ml)**	**Mean**	12.16	13.04	19.04	0.771
	**Standard deviation**	6.41	6.59	8.48	

## Discussion

It was found in the present study that children in the ADHD (93.33%) group were more likely to lack vit D than their normal counterparts (66.66%). According to a study of 1331 ADHD patients and the same number of controls aged under 18, only 8.15% of ADHD patients had normal serum vit D levels (24). In another study, the prevalence of vit D deficiency in patients with ADHD over the age of 16 has been reported to be 27% in New Zealand (25). A notable finding is that, while a high percentage of ADHD (93.33%) children were vit D deficient, healthy (66.66%) children were also found to be deficient in the vitamin. Worldwide, vit D deficiency is a problem. A high proportion of people in countries located around the Persian Gulf lacks vit D despite the abundance of sunlight. According to a study conducted in Iran and Saudi Arabia, 70% of very young girls suffer from vit D deficiency (26). Even though sunlight plays a role in maintaining vit D levels in the blood, it is insufficient to treat vit D deficiency.

In our study, serum vit D levels were significantly lower in the case group than in the control (healthy) group. The mean serum vit D levels of children aged 7 to 18 years were significantly different between cases (20.9±19.4 ng/ml) and control groups (34.9±15.4 ng/ml) in a similar study conducted in Turkey (27). According to a study of 1331 ADHD cases and the same number of controls aged under 18, ADHD children had significantly lower serum vit D levels than controls (23.5±9.9 ng/ml) (24). In contrast, vit D levels and certain behavioral problems, including ADHD, were not significantly associated in another study in England (28). Also, according to the results of an interventional study in New Zealand conducted on 80 adults with ADHD older than 16 years old, the treatment of these patients with vit D supplements for eight weeks alleviated their symptoms. However, adding other micronutrients such as zinc, vitamin B12, iron, and folate did not have the desired effect (25). Vitamin D appears to regulate nerve cell development and function (15). 

Additionally, our results indicated that there was not any significant relationship between the mean serum levels of vit D and the subtype of ADHD, but there was a statistically insignificant relation between vit D levels and the severity of ADHD, as serum levels of vit D were lower in patients with more severe diseases. The presence of 25(OH) D3-hydroxylase in the brain (which is responsible for the active form of vit D) as well as vit D receptors (20) support the notion that vit D is involved in the functioning of the central nervous system. These brain regions are associated with the development of ADHD. It has also been noted that vit D as a hormone plays a role in the synthesis of serotonin (9), a neurotransmitter involved in brain function (28). There is some evidence that adolescents with ADHD and low brain serotonin concentrations have increased aggressive behavior (29). It has also been suggested that the timing of vit D deficiency can affect cognitive and behavioral functions. During early life, low vit D concentrations may increase the risk of cognitive impairment and brain structural problems (30). Furthermore, studies have shown a 28% increase in the size of neonatal brain lateral ventricles due to vit D deficiency during pregnancy (31). The enlargement of the lateral ventricles is one of the clinical features of ADHD (32). Additionally, it has been suggested that vit D status might play a role in the dopamine system through its effect on the expression of tyrosine hydroxylase, an enzyme responsible for limiting the synthesis of dopamine (33). Polymorphisms in this enzyme's gene have been associated with ADHD in animal models (34). Serotonin and opioids, as well as dopamine, are involved in the control of mood (35). Vit D appears to regulate nerve cell development and function (15). 

As a result of the lack of significant differences in terms of age and gender between the two groups, this study has been used to eliminate bias in its results. Also, to avoid vit D bias related to sun exposure, samples were collected from June to the end of September 2020. One of the limitations of this study, is the lack of investigation and removal of the confounding factor of the amount of daily exposure to sunlight and children's BMI for a more accurate examination of the vit D serum level. In addition, this study cannot fully explain the cause-and-effect relationship due to its cross-sectional nature. In summery children with ADHD have low levels of serum vit D, suggesting a need to monitor their levels and treat patients with vit D deficiencies. A modified lifestyle and diet are also important to eliminate the nutritional deficiencies in the society.
